# Livestock across the world: diverse animal species with complex roles in human societies and ecosystem services

**DOI:** 10.1093/af/vfab047

**Published:** 2021-10-20

**Authors:** Robyn G Alders, Angus Campbell, Rosa Costa, E Fallou Guèye, Md Ahasanul Hoque, Raúl Perezgrovas-Garza, Antonio Rota, Kate Wingett

**Affiliations:** 1 Development Policy Centre, Australian National University, Canberra, ACT, Australia; 2 Kyeema Foundation, Brisbane, QLD, Australia; 3 Global Health Programme, Chatham House, London, UK; 4 Nossal Institute for Global Health, Melbourne School of Population and Global Health, University of Melbourne, Melbourne, VIC, Australia; 5 Fundação Kyeema Regional Office, Kyeema Foundation and Universidade Eduardo Mondlane, Maputo, Mozambique; 6 *Representation to the Republic of Djibouti and the Intergovernmental Authority on Development, Food and Agriculture Organization of the United Nations, Djibouti City, Republic of Djibouti; 7 Department of Medicine and Surgery, Chattogram (previously Chittagong) Veterinary and Animal Sciences University, Chattogram, Bangladesh; 8 Instituto de Estudios Indígenas, Universidad Autónoma de Chiapas, San Cristóbal de Las Casas, Chiapas, Mexico; 9 International Fund for Agricultural Development, Rome, Italy; 10 School of Life and Environmental Sciences, University of Sydney, Sydney, NSW, Australia

**Keywords:** ecosystems services, gender, livestock systems, sustainable development, therapy animals, youth

**Implications**

- Livestock are associated with multiple sociocultural and religious beliefs in societies across the globe.
- Human mental and physical health benefit from livestock services from antivenom production to therapeutic hormones to xenografts.
- Extensively raised livestock are frequently integral to the provision of ecosystem services and are an essential part of many agroecosystems and contribute to circular food systems.
- Livestock, and small livestock in particular, can contribute directly to 12 of the 17 Sustainable Development Goals.
- Moving forward, the quest is to identify and promote livestock systems that enhance environmental and human health and well-being as well as animal welfare.

## Introduction

It is anticipated that a 50% to 70% increase in food productivity will be needed to feed 9 billion people by 2050. Livestock provide for about 33% of human protein consumption and 17% of total calories (FAO, 2018). The demand for livestock products is estimated to more than double in the next 20 yr, as a result of urbanization, economic growth, and a change in consumption patterns in low- and middle-income countries (LMICs). The livestock sector represents nearly 1 billion smallholder livestock producers in developing countries and contributes 40% of agricultural Gross Domestic Product and from 2% to over 33% of household incomes. Smallholder livestock production is largely based on family farming and is key to poor rural people’s livelihoods, food security, and employment creation. Livestock provide food for household consumption, products for income generation, and quick cash when emergencies and external shocks occur (i.e., climatic conditions, diseases, and price volatility). Livestock are important assets that respond to the multiple needs of smallholders (e.g., manure, draught, and hauling power), while also having a cultural and spiritual value. Poultry and small ruminants are generally managed by and provide direct benefits to women (Guèye, 2003; FAO, 2020; IFAD, 2020).

Humans and our domesticated animals have evolved in unison over the past 11,000 yr, with domestication frequently portrayed as a relationship delivering benefits to both partners, human and domesticate (Zeder, 2012). Domestication sites and traditional production systems have strong links with local agroecological characteristics (Hocquette et al., 2018). Endurance of traditional production systems has also been closely linked with the maintenance of the health of the local environment by Hocquette et al. (2018).

Unsustainable and/or inappropriate management of livestock has contributed to environmental degradation, loss of biodiversity, emerging infectious zoonotic disease, and increased emission of greenhouse gases (van Zanten et al., 2019). These impacts have increased in severity in line with increasing human population, purchasing power, and urbanization since the mid-20th century (Thornton, 2010).

This paper provides a high-level overview of the huge array of contributions made by livestock to human society beyond the provision of food and fiber with a focus on terrestrial, vertebrate species. With a focus on LMICs, we stress the significant role of livestock in sustainable development, including: ecosystems services via nature-based livestock production systems; sociocultural and religious roles; and, contributions to human health and well-being beyond animal-source food.

## Variations in Livestock Production Systems

Livestock production varies widely across the globe in terms of species raised, the purposes for which livestock are kept, the scale and intensity of livestock production systems, the agroecologies where the systems occur, and the resources they consume. Livestock production has also changed rapidly in recent decades, due to increasing demands for animal products, population growth, and greater incomes, urbanization, and changing livestock production technologies (World Bank, 2009). In addition to being kept to produce various products (meat, milk, fiber, hides, eggs, and manure for fertilizer or fuel), livestock are still frequently kept to provide draught power and transport, as a social or cultural asset, and as a financial asset—particularly where financial systems are underdeveloped or inaccessible (Wilson, 2009; Acosta et al., 2018). These uses are discussed in more detail in subsequent sections.

**Figure 1.**
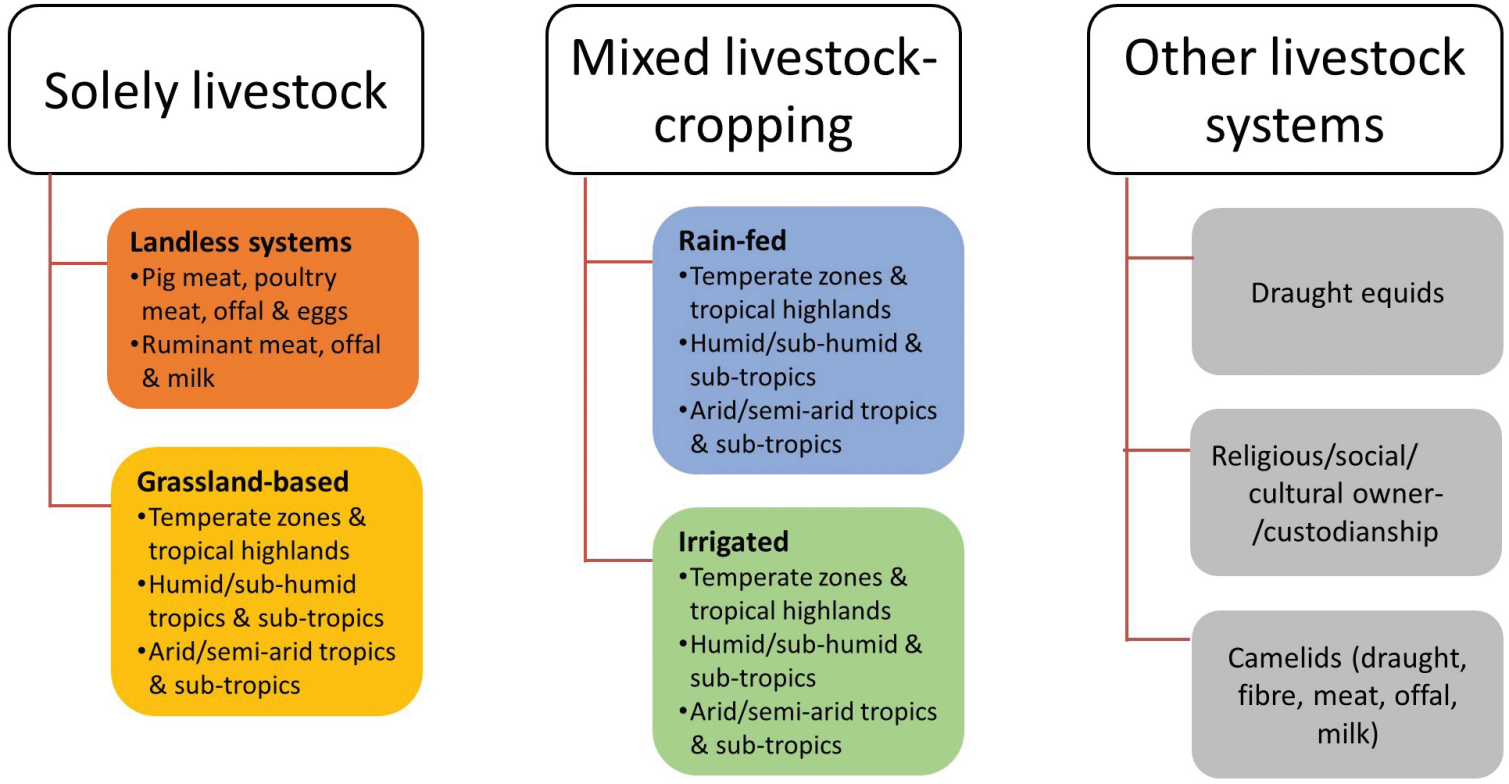
Classification of livestock production systems, adapted from Steinfeld et al. (2006).

Classification of livestock systems may be based on land use, agroecological zone, the integration of livestock with cropping systems, the intensity of production, and the kind(s) of products produced (Steinfeld et al., 2006), although it tends to mainly rely on land use, agroecological zone, and integration with cropping (Seré et al., 1996; Figure 1).

*Solely livestock systems* generate a majority of income directly from livestock, with the majority of animal feed directly grown or sourced for livestock, rather than from the system’s crop-growing activities (Seré et al., 1996). Within this category, *grassland-based* systems produce more than 10% of consumed dry matter on the farm and have stocking densities of less than 10 livestock units per hectare of agricultural land. These systems occupy 26% of the earth’s ice-free land surface (Steinfeld et al., 2006). Grassland grazing systems typically occur where topography, low temperature, or low rainfall limits the ability to grow crops. Examples of these production systems include herding on the Mongolian steppe; cattle ranching in the west and central Africa, and South America; hill country sheep farming in New Zealand; and sheep and cattle farming in inland northern Australia (Thornton et al., 2002).

In contrast, *landless solely livestock* systems produce a minority of feed locally and have much greater stocking densities. They include ruminant, pig, and poultry production systems. More than half of global pork production and 70% of global poultry meat production come from landless (referring to the livestock production units and not the crops required to feed them), intensified systems, approximately evenly divided between higher-income countries and lower- to middle-income countries (Steinfeld et al., 2006). Ruminant fattening enterprises in the Middle East are another example of intensive landless systems (Kruska et al., 2003).

Complex factors influence where landless, intensified livestock production systems are located, including access to input and output markets, especially cereal grains for use as feed, proximity to large population centers, land price, and infrastructure and storage facilities (Robinson et al., 2011). Conversely, landless systems may be geographically remote from these factors, as intensification is also driven by relative prices of animal feeds vs. animal products, and access to crop, livestock and food production, storage, and transport technologies.

In areas favorable for crop production, livestock may be mixed with rainfed or irrigated cropping systems. In *mixed livestock-cropping* systems, a majority of the livestock diet comes from cropping activities on the farm, and non-livestock farming constitutes a greater proportion of the total value of farm production (Seré et al., 1996). Examples of mixed livestock-cropping systems include buffalo-rice production in Southeast Asia, farming small ruminants for meat and fiber in the Central Dry Zone of Myanmar (Win et al., 2019), and dairy, beef, and wool- and meat-sheep production in southern Australia.

In general, the density of livestock tends to increase from grazing, to mixed rainfed and irrigated, to landless systems. There are a variety of interdependencies between different kinds of livestock systems, other agricultural enterprises, and land uses. For example, animals may be moved between different livestock systems, with breeding and rearing taking place in one system and other production, such as fattening, occurring elsewhere; this may range from a routine, systematic activity to a haphazard practice affected by markets or seasons (Nozières et al., 2011). Substantial inter-relationships occur between livestock and non-livestock activities in mixed systems beyond the provision of feed, as livestock may support crop production with draught power, transport, and manure. Additionally, livestock may have substantial positive or negative effects on biodiversity, and provisioning and regulating ecosystem services that support other agricultural and livelihood activities (Baumung et al., 2018), and economically complement other agricultural activities, including other livestock enterprises, by diversifying household income and supporting economic security (Acosta et al., 2018).

## Sociocultural and Religious Values

**Figure 2.**
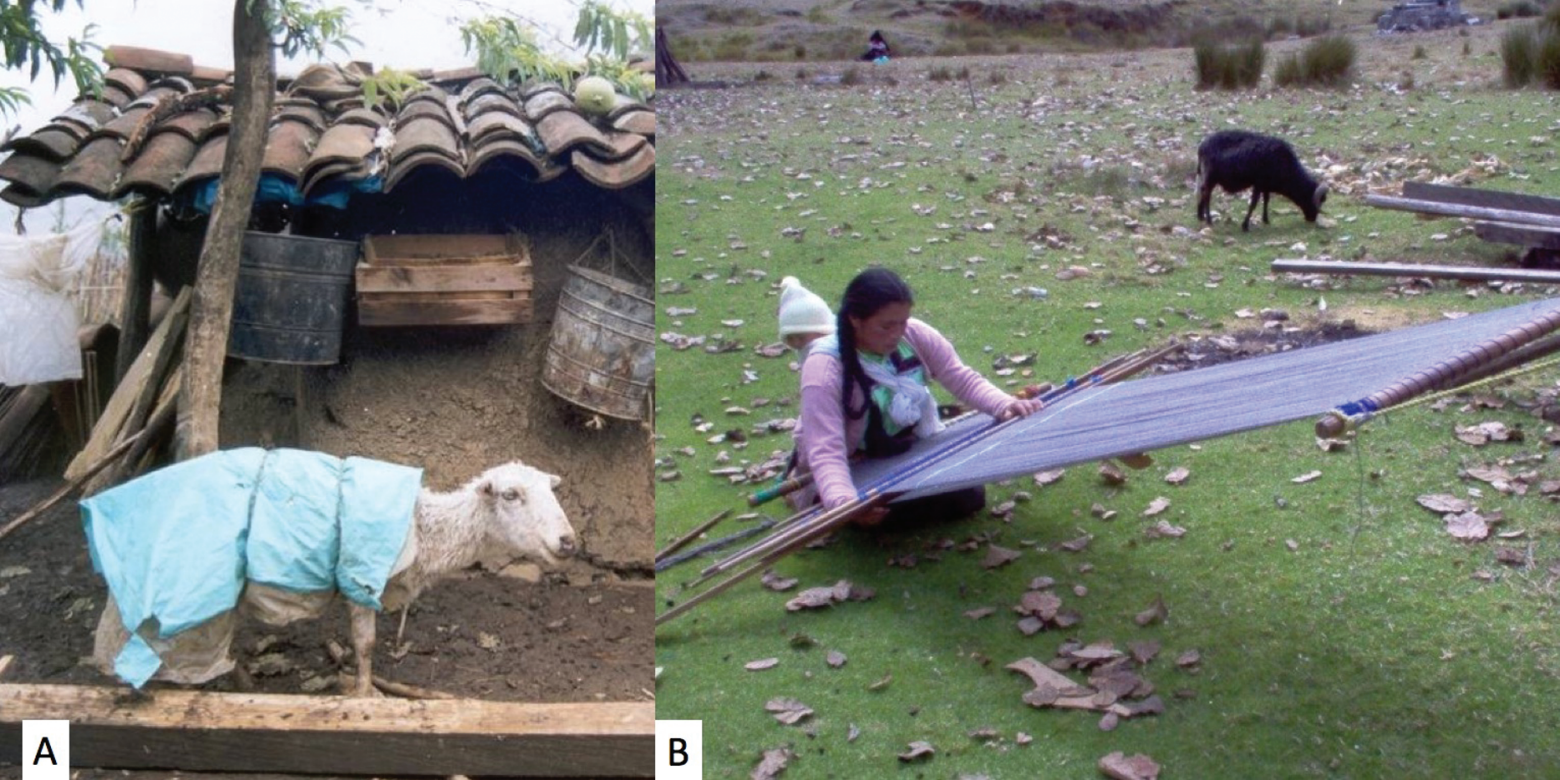
(A) Chiapas Sheep breed cared for by Tzotzil shepherdesses; sociocultural beliefs give sheep an important value as part of the family: given names, prohibition to kill them or harm them, individual caring, and constant prayers to keep them healthy; (B) Mayan Tzotzil weaver transforming wool into traditional garments, culturally preserving both identity strategies (typical textiles and weaving techniques) and local Chiapas sheep; these animals have been selected by women over centuries to produce fleeces with specific characteristics (double-coated fleece, staples with more abundant long-coarse primary fibers, and less proportion of short-thin secondary fibers) (Credit: R.P.-G.).

Sociocultural and religious values of livestock are frequently important in communities that have raised livestock over many generations. For example, in the Hindu religion, the cow is considered a sacred symbol of life to be protected and revered as are sheep in the Chiapas Region of Mexico (Figure 2). In some cultures, sacrificing animals to God or deities is performed to obtain blessings or favors. In southern Bhutan, for example, it has been reported that poultry play an important role in the worship of deities. It is believed that the deities require animals be offered in pairs; a chicken, duck, or pigeon can be paired with a large animal or can be offered in the place of a goat or pig. Farmers in the region believe that the offerings will ensure that there will be no sickness in their household (Hilmi et al., 2011). Nutrition research in Timor-Leste has revealed that animal-source food consumption is closely linked with the number of cultural ceremonies and this has the potential to make a significant difference to household diets where maternal and child malnutrition is high (Wong et al., 2018). In West Africa, there is a large demand for small ruminants. For certain populations, goat meat is a delicacy, whereas for others such as the Kpa Mende tribe in Sierra Leone preference for goat is related to secret society purposes. Among the Muslim population, sheep, particularly rams, have gained preference for carrying out many Islamic religious feasts (i.e., naming ceremonies, *Tabaski* celebration–the feast of sacrifice). *Tabaski* or *Eid al-Adha* is the biggest public holiday in Senegal, Guinea, Mali, and many other countries in West Africa. Around four million sheep are slaughtered every year for *Tabaski* in Senegal alone. In general, non-Muslim populations have kept a richer collection of cultural practices around livestock, associated with sacrifices or celebrations of the agricultural calendar (FAO, 2020).

Certain restrictions or bans regarding the rearing of certain animal species and the consumption of their food products exist in some traditional local communities (Guèye, 2003; Meyer-Rochow, 2009). Although such sociocultural constraints on livestock rearing are declining, several examples continue to be reported. People in some African communities are reluctant to keep ducks and to consume their products, as these birds are considered dirty and/or destructive to the water supplies, and/or are seen as associated with evil omens (Guèye, 2003). In Senegal, Guinea fowl rearing is considered by some communities as being a sign of poverty, whereas in other communities, Guinea fowls are regarded as good omen.

## Contributions to Human Health and Well-Being

**Figure 3.**
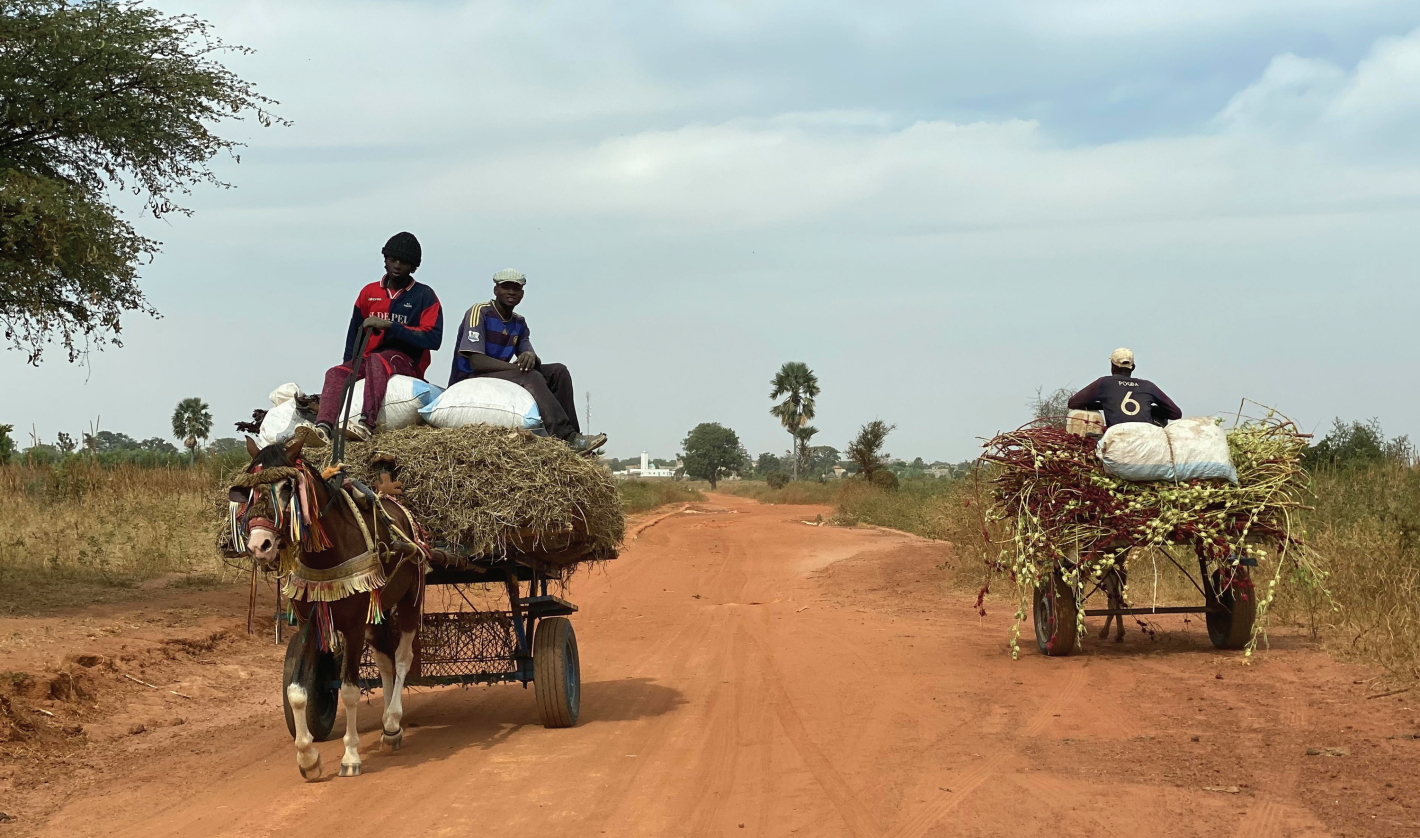
Horse transporting hay as animal feed near Ngouye, Kaffrine Region, Senegal, in November 2020 (Credit: E.F.G.).

Livestock have been demonstrated to enhance human mental, physical, and spiritual health and well-being in a vast range of ways. Scanes (2017) documents four key contributions not always sufficiently recognized: 1) production of immunoglobulins for use as anti-sera—livestock, and horses, in particular, are used to produce a range of antivenoms and antitoxins; 2) use of animal hormones and xenografts—the most notable example of hormone treatment is the use of insulin from cattle and pigs in the control of diabetes. Heart valves from cattle and pigs have been used for several years as replacements for problematic human valves; 3) service and therapy animals—livestock are used as service animals (e.g., horses in the defense services) and as therapy animals; and 4) draught power—animals such as buffaloes, camels, cattle, donkeys and horses continue to provide draught power across Africa, South and Southeast Asia, and Latin America (Figure 3).

The role of livestock, and cattle in particular, in spiritual health is a feature of the Hindu religion. The Hindus believe that a person is what he or she eats. Food prepared from the products of a *sattvik* (virtuous) cow is thought to bring good and positive energy to the body and help people to gain happiness. Hindus believe that cow’s milk can transform the mindset from negative to positive. The milk of an Indian cow is considered as very divine and *sattvik* (virtuous), as it is known to attract the divine vibration or Shakti (Korom, 2000).

Human health research also benefits from livestock. Livestock animal models have also been used to improve prevention and therapeutics for humans in the area of microbiology and infectious disease (transmissible spongiform encephalopathies, mycobacterial infections, influenza A virus infection, vaccine development and testing, smallpox vaccine, and the human microbiota) and metabolic, neoplastic, and genetic disorders (stem cell therapy, male germ line cell biology, pulmonary adenocarcinoma, muscular dystrophy, and wound healing (Roth, 2011; Lairmore and Khanna, 2014).

Personal and intimate contact with farm animals has been reported to enhance the physical, social, and mental health of people with diverse needs (Hodges, 1999; Hassink et al., 2017; Scanes, 2017; Cacciatore et al., 2020). It has been suggested that contact with farm animals can provide relaxation and reduce stress and anxiety, provide meaningful day occupation and distract people from negative emotions, facilitate interpersonal relations, and provide social support (Hassink et al., 2017; Table 1).

**Table 1.** Perceived benefits of farm animals for different types of participants

Meaningful day occupation	Ordinary work, purposeful tasks, meaningfulness
Valued relationship	Appreciation, closeness, warmth, safe, trustful, relationship without stigmatization or complications, social support, something to engage with, living creature to tell stories to
Mastery of tasks	Ability to accomplish work tasks, building motivation and confidence, responsibility, conquer of fear
Reciprocity	Giving care to other living beings, becoming caregiver
Distraction from problems or difficulties	Screening out negative perceptions, getting energy
Relaxation	Tranquillity, feeling comfortable
Tailored care/support	Working at your own pace, choice in activities
Relationship with other human beings	Shared relations, knowledge and experiences, stimulating conversation
Stimulating health behavior	Physical and healthy activity in implicit way
Welcoming environment	Feeling at home, place attachment

Source: Hassink et al. (2017).

These contributions of livestock and interactions between livestock and farming households can still be seen, in rural areas of Africa, Asia, and Latin America. Here, one can also see the vital role of domestic animals in permitting rural people to survive and to maintain human dignity in the current conditions of great poverty. Although livestock ownership fulfills important beneficial ancillary roles in people’s lives, it can have adverse effects in certain situations. For example, poor mental health has been reported in farmers facing adverse events that are left unaddressed, such as disease outbreaks, drought, and conflict causing loss of livestock (Nuvey et al., 2020).

## Circular Food System Contributions

It is now widely acknowledged that the global food system is having major detrimental impacts on the environment (FAO, 2018; van Zanten et al., 2019). The traditional “take–make–dispose” approach of linear economies, where raw materials are collected, then transformed into products that are used until they are finally discarded as waste, is showing its limits. Circular economies offer a different vision and the 3R approach: “reduce (use of natural resources), reuse, and recycle” (Milios, 2018) seems to move in the right direction of an increased eco-effectiveness of production systems. As highlighted above, there are many different livestock production systems, and some do contribute positively to sustainable food systems when reared under a circular paradigm.

**Figure 4.**
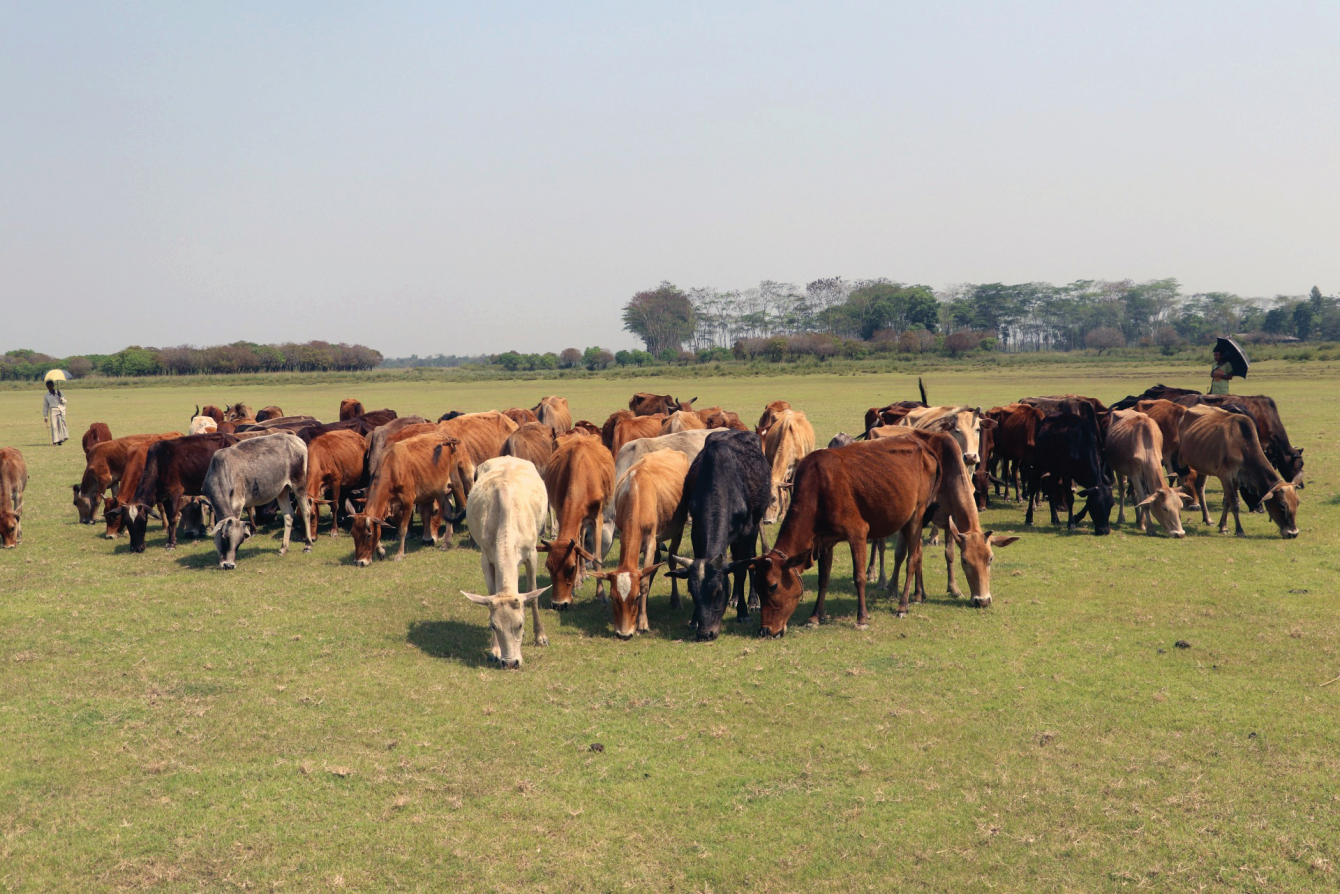
Indigenous cattle reared under extensive rearing system, grazing on the field in Sylhet areas of Bangladesh (Credit: Shuvo Singh).

Under circular production systems, arable land is primarily used for crop production with biomass unsuitable for direct human consumption being recycled as animal feed. Crop byproducts, crop residues, failed crops, and food waste may be used as valuable components in the diets of livestock. Chemical residue and disease risks must be managed when these strategies are employed in feeding food-producing animals (NSW Department of Primary Industries, 2019). Grasslands are used for extensively raising livestock, converting grasses and legumes that are inedible for people into nutrient-dense food and natural fiber (Figure 4). Crop byproducts, livestock manure, and treated food waste and human excreta are used to maintain soil fertility. In this way, nutrients are recycled, and livestock contribute to a circular food system that sustainably nourishes future generations (van Zanten et al., 2019). In this context, working animals would have an important role, especially for smallholder producers, particularly those located in remote areas and/or with difficult access (e.g., mountain areas). Another example of the role of livestock in circular bioeconomies is the production of biogas from intensive piggery manure ponds. This reduces environmental carbon emissions from the manure while simultaneously reducing the need to burn fossil fuels for energy production (Australian Pork Limited, 2021). Through reusing and recycling byproducts and waste of food and natural fiber systems on extensive and intensive livestock enterprises, fewer virgin natural resources are required as inputs to the food system. These actions reduce the environmental impact of animal-source food value chains and increase their gross profit margins.

## Ecosystem Services Contributions

Ecosystem services are the multiple benefits to humans (e.g., food, clean water, shelter, and raw materials for our basic needs) provided by healthy ecosystems, including agroecosystems, forest ecosystems, grassland ecosystems, and aquatic ecosystems (FAO, 2016).

Livestock are frequently integral to the provision of ecosystem services and are an essential part of many agroecosystems. Their roles in these systems include: 1) transforming feed inedible by humans into nutritious foods (Figure 4), useful products such as pharmaceuticals and companion animal feed, fuel (through manure), and transport; 2) enhancing ecosystem health through grazing, browsing, trampling, and the production of dung and urine; and 3) shifting locations allowing them to respond to fluctuations in resource availability and weather patterns.

Well-managed livestock production systems can contribute to increasing agro and environmental land cover and biodiversity (FAO, 2016). In addition, carbon-neutral and economically profitable livestock farming is achievable with the correct balance of trees, grassland, and livestock stocking density (Doran-Browne et al., 2016). Other benefits of sustainable livestock farming include limiting soil erosion and water evaporation and providing habitats for a wide variety of invertebrate and vertebrate species (Brandle et al., 2004).

With increased frequency of wildfires in many locations in the 21st century, grazing and browsing livestock can assist with reducing fuel loads (Leroy et al., 2018).

## Role in Sustainable Development Goals, Including Empowering Women and Youth

**Figure 5.**
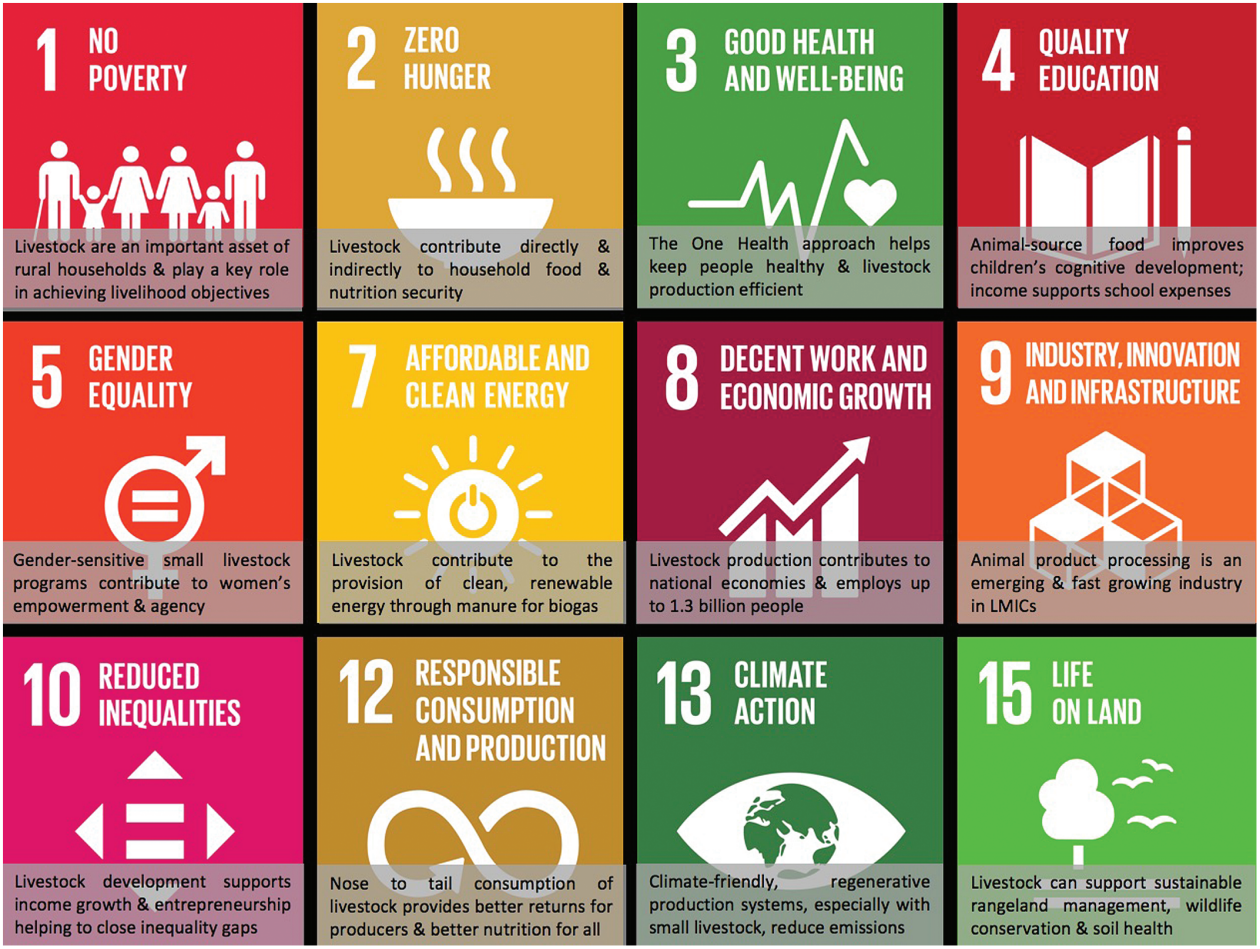
Well-managed livestock production systems have the potential to contribute to 12 of the 17 SDGs, especially in countries with a long history of livestock production (adapted from IFAD, 2020).

**Figure 6.**
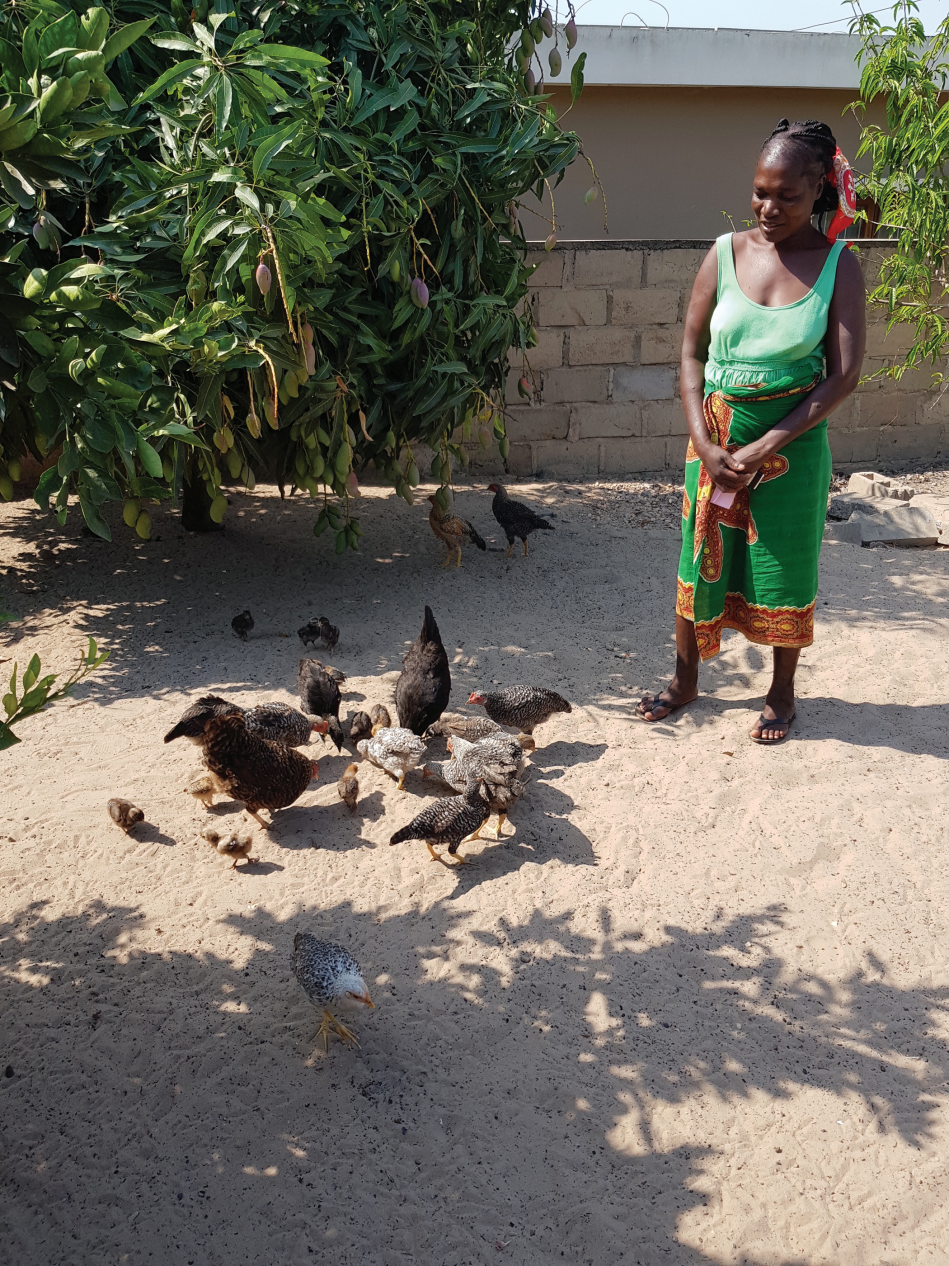
Luisa Julião Mazive a widow from Marracuene District in Mozambique received four indigenous village chickens (3 hens and 1 rooster) in April 2018 and by December 2020 she had 57 chickens. During this time, she has vaccinated the birds regularly against Newcastle disease and directly benefited from the regular sale and home consumption of birds, saying that “I am very satisfied with the support given to me, my life has improved considerably.” (Credit: R.C.).

In 2015, all Member States of the United Nations signed up to achieving the Sustainable Development Goals (SDGs) by 2030. Livestock production makes a major contribution to national economies worldwide. Globally, up to 1.3 billion people are employed in different livestock product value chains and half of the world’s 900 million poor depend on livestock for their livelihoods (Keeling et al., 2019). Livestock production can boost economic growth in two main ways: through direct contribution to rural livelihoods and agricultural output, and, given the sector’s various linkages with other industries, through the multiplier effects of livestock products moving along expenditure and supply chains (FAO, 2018). Policies that promote appropriate sustainable livestock systems lead to improved animal production and welfare, higher labor productivity, and value-adding to production. Livestock, and small livestock in particular, can contribute directly to 12 of the 17 SDGs (IFAD, 2020;Figure 5). Sustainable livestock production can strengthen household livelihoods (SDG1) and improving children’s cognitive and physical development and school attendance and performance through the consumption of appropriate quantities of animal-source food (SDG4). Livestock, especially smallstock, contribute to empowering women through gender-sensitive livestock programs (SDG5; Figure 6). Sustainable and equitable livestock production can provide decent employment for young men and women (SDG8; FAO, 2018). Livestock systems involving draught and other work make essential, but often overlooked, contributions to SDGs and economic development, including in urban areas.

## Conclusions

The world is at a critical juncture as individuals and countries seek to counteract the far-reaching impacts of the climate crisis, biodiversity loss, growing inequity, emerging and reemerging infectious diseases, and noncommunicable diseases. The challenge for the livestock, public health, and environmental sectors will be to identify and promote the livestock production systems that enhance the health of people, animals, and the environment. This will also involve enabling producers to transition away from production systems that are unable to demonstrate a net positive impact.

Livestock are about so much more than food and fiber. It is, therefore, critical to understand these contributions to local cultures and ecosystems, as well as account for local perspectives, as we work together to deliver the SDGs.

## About the Authors

**Robyn G. Alders** is a Senior Consulting Fellow with the Chatham House Global Health Program and an Honorary Professor with the Development Policy Centre, Australian National University. She has worked in sub-Saharan Africa, SE Asia, and Oceania for over 30 yr. She also consults to the Food and Agriculture Organization and the International Fund for Agricultural Development. Her current research and development interests include sustainable livestock production in support of food and nutrition security, One/Planetary Health, gender equity, and science communication.

**Angus Campbell** leads the One Health Unit at the Nossal Institute for Global Health, University of Melbourne. He is a livestock veterinarian and veterinary epidemiologist, and has worked in whole-farm veterinary consultancy, veterinary training, and applied research in southern Australia and South, Central, and Southeast Asia for 20 yr. He works to improve sustainable livestock production in order to support human livelihoods, through better livestock health and management, addressing human–animal disease interaction, farm production efficiency, and evaluating animal health economics and farm profitability.

**Rosa Costa** is the Africa Regional Manager for Kyeema Foundation and, since 1986, a lecturer at the University Eduardo Mondlane. She has worked as the Director of the Mozambican National Veterinary Research for more than 20 yr. From 2009 onwards, she has coordinated the implementation of food security and capacity building projects in member states of the African Union focussing on the sustainable control of Newcastle disease in chickens in the family sector. She has worked in Mozambique, Tanzania, Malawi, Zambia, Angola, and Ethiopia. In 2015, she became a member of the Kyeema Board and is a current member of the Program Monitoring and Evaluation (PME) Sub-committee of the Board.

**E. Fallou Guèye** is an International Project Coordinator at the Food and Agriculture Organization of the United Nations (FAO) Representation to the Republic of Djibouti and the Intergovernmental Authority on Development (IGAD), Djibouti. For 30 yr, he has been working on livestock-centered poverty alleviation, food security, and strengthening of sustainable livelihoods for the benefit of resource-poor and vulnerable communities by promoting capacity building for stakeholders, advocacy for policy makers, and enhancement of institutional environments. His areas of work include emergency/post-emergency, rehabilitation, research, and development situations. He has also been a resource person for various scientific institutions and bodies devoted to animal agriculture research and development. He is the author of numerous scientific articles, book chapters, workshop papers, and congress proceedings.

**Md. Ahasanul Hoque** is a Professor of Epidemiology at Chattogram Veterinary and Animal Sciences University in Bangladesh. He is the National Coordinator of the UKRI-funded One Health Poultry Hub, an OIE-funded Veterinary Education Twinning Project, and a co-investigator on the Swedish Research Council-funded Climate change mitigation through a sustainable water buffalo dairy chain in Bangladesh. He is the author of numerous national and international scientific publications.

**Raúl Perezgrovas-Garza** is a full-time researcher and lecturer at the Instituto de Estudios Indígenas, Universidad Autónoma de Chiapas, México. He has a PhD in Agroecology and Sustainable Development, University of London, UK. He has been working at the Universidad Autónoma de Chiapas for over 35 yr, mostly as a researcher with interests in traditional agricultural systems, social and environmental implications of locally adapted animal breeds, and history of domestic animals in America.

**Antonio Rota** is the IFAD Lead Technical Specialist in livestock development. He is responsible for providing technical advisory services on livestock and pastoral systems issues. He advocates for inter-institutional and cross-regional exchange of knowledge to enhance nutrition and gender-sensitive, climate-resilient, and sustainable intensification of smallholder livestock production. He has 35 yr of professional experience in the livestock development sector. Before joining IFAD, he worked with FAO, NGOs, and private consulting firms in sub-Saharan Africa, Asia, Latin America, and the Caribbean. With FAO, he operated as Animal Production Officer for the Caribbean and Senior Technical Adviser in Afghanistan with the responsibility of implementing FAO projects on rural poultry production and development of integrated dairy schemes.

**Kate Wingett** is a PhD candidate at the University of Sydney. She worked as a private practitioner veterinarian for 19 yr in Australia and the United Kingdom and is now a government veterinary policy officer. Currently, she is completing her PhD on the role ruminant production systems have in achieving food and nutrition security, with a focus on monitoring production and consumption of products from these value chains.
